# Synergistic efficacy of combined neurolysis and methylcobalamin in peripheral nerve injury: a randomized clinical trial

**DOI:** 10.3389/fnint.2026.1747898

**Published:** 2026-04-08

**Authors:** Xuwei Hu, Zuoshi Li

**Affiliations:** Department of Neurosurgery, Jieyang People’s Hospital, Jieyang, Guangdong, China

**Keywords:** disease, inflammation, methylcobalamin, neurolysis, peripheral nerve injury

## Abstract

**Introduction:**

Peripheral nerve injury (PNI) imposes significant burdens, requiring therapies targeting both mechanical compression and inflammatory pathophysiology. While neurolysis addresses extrinsic compression and methylcobalamin promotes intrinsic nerve repair, their combined potential is underexplored. This study compared the efficacy of neurolysis monotherapy, methylcobalamin monotherapy, and combination therapy across functional, electrophysiological, and inflammatory outcomes in PNI.

**Methods:**

Ninety PNI patients were randomized to three groups (*n* = 30/group): neurolysis alone, methylcobalamin alone (0.5 mg tid), or combination therapy. Outcomes at 60 days comprised functional recovery (Carroll Scale), nerve conduction velocities (MCV, SCV, AMP, LAT), joint mobility (AROM/PROM), pain severity (Global Pain Scale), and serum cytokines (NF-κB, TNF-α, IL-6 via ELISA).

**Results:**

The combination group demonstrated significantly higher total therapeutic efficacy (86.67%) compared to neurolysis alone (50.00%) and methylcobalamin alone (53.33%; *P* < 0.05). All groups showed significant post-treatment improvements in nerve conduction velocities, joint mobility parameters, pain scores, and inflammatory cytokine levels (*P* < 0.05). However, combination therapy yielded superior outcomes: it produced the greatest improvements in active and passive range of motion, the most significant reductions in pain scores across all assessed domains, and the lowest post-treatment levels of inflammatory cytokines (NF-κB, TNF-α, IL-6; *P* < 0.05 versus either monotherapy).

**Discussion:**

The combination of neurolysis and methylcobalamin synergistically enhances functional recovery, nerve conduction, joint mobility, pain relief, and anti-inflammatory effects in PNI, demonstrating superior clinical efficacy compared to either treatment administered alone.

## Introduction

Peripheral nerve injury (PNI) constitutes a major clinical burden, arising from diverse origins including trauma, compression syndromes, metabolic disorders, and inflammation, with traumatic injuries alone affecting an estimated 13–23 individuals per 100,000 population annually (Robinson, 2022; Pitman et al., 2025). These injuries frequently lead to chronic pain, sensory-motor deficits, significant disability, and substantial socioeconomic burdens. The ensuing pathophysiology involves structural compromise (Wallerian degeneration, axonal disruption, demyelination, perineural fibrosis/adhesions/compression) alongside a detrimental inflammatory cascade, where key mediators like NF-κB, TNF-α, and IL-6 perpetuate nerve damage and impede regeneration (Liu and Duan, 2023). Consequently, effective therapeutic interventions must concurrently address both the mechanical impediments to nerve function and the hostile biochemical microenvironment hindering repair (Modrak et al., 2020).

Current management approaches for PNI often involve surgical intervention or pharmacological neuroprotection/regeneration strategies (Lopes et al., 2022). Surgical neurolysis aims to directly alleviate mechanical compression and entrapment by meticulously releasing the nerve from constrictive scar tissue, adhesions, or fibro-osseous tunnels, thereby restoring gliding mobility and blood supply (Sabourin et al., 2022). While potentially effective in decompressing the nerve, surgery alone may not fully address the intrinsic biochemical derangements affecting neuronal recovery (Kong et al., 2022). Pharmacologically, methylcobalamin (the active form of vitamin B12) has demonstrated neurotrophic properties, promoting axonal regeneration, myelin synthesis, and neuronal cell survival (Zhang et al., 2020). It is widely utilized as a neuroprotective agent in peripheral neuropathies (Ramadhani et al., 2024). However, monotherapy with methylcobalamin may be insufficient for injuries requiring structural decompression.

Given the complementary mechanisms of action between neurolysis and methylcobalamin, a combined therapeutic approach holds strong biological plausibility for superior outcomes in PNI. Neurolysis addresses extrinsic compression and facilitates a conducive mechanical environment (Goncalves et al., 2023), whereas methylcobalamin promotes intrinsic neuronal repair and modulates inflammation (Ji et al., 2022). Despite the clinical use of both modalities, either alone or empirically in combination, robust comparative evidence evaluating their individual and synergistic efficacy across multiple functional, electrophysiological, and biochemical parameters remains relatively limited.

Therefore, this study was specifically designed to conduct a systematic comparison of the therapeutic efficacy of surgical neurolysis alone, methylcobalamin monotherapy alone, and the combination of neurolysis with methylcobalamin in patients with PNI. The primary objective was to rigorously assess and contrast the functional recovery, nerve conduction parameters, joint mobility, pain relief, and modulation of key inflammatory markers achieved by these three distinct treatment strategies. This investigation aims to provide critical evidence to inform optimal clinical decision-making for the management of peripheral nerve injuries.

## Materials and methods

### Clinical data

The sample size was determined *a priori* based on a power analysis detailed below. A total of 90 patients with PNI admitted to Jieyang People’s Hospital were enrolled in this study. In this study, PNI was specifically defined as a predominantly focal, mechanical nerve lesion resulting from trauma (e.g., crush, traction, laceration) or chronic compression/entrapment (e.g., carpal tunnel syndrome, cubital tunnel syndrome), confirmed by clinical examination and electrophysiological studies. Using a random number table, participants were randomly allocated into three treatment groups (*n* = 30 per group): neurolysis alone, methylcobalamin alone, or neurolysis combined with methylcobalamin. No significant differences were observed among the three groups regarding baseline characteristics, including gender, age, or body mass index (BMI) (all *P* > 0.05).

The study protocol received approval from the Hospital’s Medical Ethics Committee. Inclusion criteria were: (1) Definite diagnosis of traumatic or compressive PNI confirmed by relevant examinations upon admission; (2) Complete baseline data and good treatment compliance enabling active cooperation; (3) Symptom duration > 10 days; (4) Age ≥ 20 years; (5) Provision of written informed consent by all patients and their family members; (6) Compliance with ethical principles for medical research. Exclusion criteria included: (1) Altered mental status (e.g., coma or stupor); (2) Poor treatment cooperation or compliance; (3) Significant dysfunction of vital organs; (4) Presence of concomitant injuries; (5) Comorbid mental disorders or a history of psychiatric illness; (6) Presence of systemic diseases known to cause polyneuropathy (e.g., diabetes mellitus with HbA1c > 7.0%, active malignancy undergoing chemotherapy, chronic renal failure, autoimmune disorders); (7) History of toxic exposure leading to neuropathy; (8) Diagnosis of generalized polyneuropathy of any cause.

### Ethical considerations and patient safety

Stringent measures were implemented to safeguard patient interests. Surgical indications were rigorously reviewed by a multidisciplinary committee, requiring objective evidence of nerve compression and failure of conservative therapy. A dedicated safety monitoring board conducted regular reviews, with a protocol for immediate intervention upon any clinical deterioration. All participants received active, protocol-driven treatment and rehabilitation, ensuring that trial participation provided structured care meeting or exceeding standard practice.

### Sample size estimation

The sample size was calculated *a priori* using G Power software (version 3.1.9.7). Based on preliminary data, we estimated that the combination therapy would yield a large effect size (*f* = 0.40) on the primary outcome of functional recovery (Carroll Scale score) compared to either monotherapy. For a one-way analysis of variance (ANOVA) comparing three independent groups, with an alpha level of 0.05 and a desired statistical power of 80%, the minimum required sample size was calculated to be 66 (22 per group). To account for a potential attrition rate of approximately 10%, we aimed to enroll at least 30 patients per group, resulting in a total sample size of 90 participants. This sample size provides adequate power to detect significant between-group differences in the primary and key secondary outcomes.

### Randomization and blinding

This study was designed as a prospective, randomized, evaluator-blinded clinical trial. An independent statistician not involved in patient recruitment or treatment generated a computer-based random number sequence using a permuted block design (block sizes of 6) to ensure balanced allocation. Allocation concealment was implemented by placing group assignments in sequentially numbered, opaque, sealed envelopes, which were opened only after the patient had completed all baseline assessments and just before the assigned intervention. Participants were randomly assigned in a 1:1:1 ratio to one of three treatment groups: neurolysis alone, methylcobalamin alone, or neurolysis combined with methylcobalamin. Owing to the nature of the surgical intervention (neurolysis), blinding of patients and surgical personnel was not feasible. However, to minimize assessment bias, all outcome evaluations—including functional assessment (Carroll Scale), nerve conduction studies, range of motion measurements, pain scoring (Global Pain Scale), and laboratory analyses (ELISA for inflammatory cytokines)—were performed by independent assessors who were blinded to the group allocation and had no access to the randomization list. Data entry and statistical analyses were also conducted by personnel unaware of treatment assignments, and the allocation code was broken only after the final statistical analysis was completed. These measures were taken to reduce potential performance and detection bias.

### Treatment method

#### Neurolysis treatment

To minimize surgical heterogeneity, all neurolysis procedures were performed by the same two experienced neurosurgeons following a standardized surgical checklist. The surgical approach, extent of decompression, and criteria for determining adequate release were strictly predefined and adhered to for every patient. Under either fluoroscopic guidance or intraoperative neuromonitoring (Nicolet Endeavor CR, Natus Medical Inc., United States), the affected nerve segment was meticulously exposed. A longitudinal incision along the course of the nerve was made to access the compression site. Circumferential adhesiolysis was performed for a minimum of 2 cm proximal and distal to the identified pathology (e.g., scar band, osteophyte, or fibrotic tunnel) to ensure adequate decompression and gliding. Adhesions and constrictive scar tissue were released using microsurgical techniques, with care taken to preserve the epineural vasculature. For cases involving entrapment within fibro-osseous tunnels, a minimal but complete decompression was performed. Intraoperative confirmation of adequate decompression was assessed by visual inspection of restored nerve gliding and, when applicable, by direct observation of improved intraoperative nerve action potential amplitudes. The need for epineurial or fascicular suture was predetermined based on preoperative electrophysiological and imaging (ultrasonography or MRI) evidence of nerve transection or severe axonal disruption; all such repairs were performed using 9–0 or 10–0 nylon sutures under an operating microscope. All procedures concluded with copious irrigation and meticulous hemostasis before layered closure.

#### Rehabilitation protocol

A standardized, protocol-driven rehabilitation program was implemented for all patients. Initiated on the third postoperative day for surgical groups and similarly recommended for the methylcobalamin-only group, it consisted of two phases: (1) Early phase (Days 3–28): edema control, passive range of motion exercises performed by a certified therapist twice daily, and isometric muscle activation. (2) Late phase (Day 29 onward): active-assisted and active range of motion exercises, sensory re-education, and light resistance strengthening. Rehabilitation adherence was monitored through therapy logs. To ensure consistency across groups, all patients received rehabilitation instructions from the same team of physical therapists, and compliance was documented in standardized logs reviewed weekly by an independent coordinator. Patients in the methylcobalamin-only group were provided with the same structured rehabilitation plan and were instructed to perform the exercises at home, with adherence verified through telephone follow-up every 3 days.

#### Methylcobalamin treatment

Patients received oral methylcobalamin tablets (Hangzhou Conba Pharmaceutical Co., Ltd.; Approval No.: H20060921; Strength: 0.5 mg/tablet) at a dosage of one tablet three times daily (tid).

#### Neurolysis combined with methylcobalamin treatment

Patients underwent the standardized neurolysis surgical procedure as described above, followed by concomitant administration of the conventional methylcobalamin regimen (one tablet orally, three times daily). All three treatment groups received therapy for 30 days as one treatment course, with two consecutive courses administered.

### Efficacy evaluation

The primary outcome was the change in the Carroll Upper Extremity Function Test score (a continuous scale from 0 to 99, with higher scores indicating better function) from baseline to 60 days post-treatment initiation. Functional outcomes were graded as follows: Grade I-II (score < 50): very poor function; Grade III (score 51–75): poor function; Grade IV (score 76–89): incomplete function; Grade V (score 90–98): complete function.

As a secondary outcome, therapeutic response was categorized based on the change in the Carroll score from baseline to day 60, using the following objective and reproducible criteria: Cured: Carroll score of 99 (normal function). Markedly Effective: Improvement of ≥ 2 grades on the Carroll scale (e.g., from Grade II to Grade IV or from Grade III to Grade V). Effective: Improvement of 1 grade (e.g., from Grade III to Grade IV). Ineffective: Any improvement in score that did not result in a grade advancement, no change in score, or a decreased score. The overall response rate was then calculated as the percentage of patients in each group classified as “Cured,” “Markedly Effective,” or “Effective.” This dichotomous outcome complements the primary continuous analysis of the Carroll score change.

### Nerve conduction studies

A comprehensive electrophysiological assessment was performed for all three groups before and after treatment using a Viking Quest electromyography/nerve conduction study system (Nicolet Biomedical Inc., United States). The evaluated parameters included: (1) Motor nerve conduction velocity (MCV) and sensory nerve conduction velocity (SCV) to assess the speed of impulse propagation; (2) Electromyography latency (LAT) of the compound muscle action potential, indicating the efficiency of the fastest-conducting fibers; and (3) Amplitude (AMP) of the compound muscle action potential, reflecting the number and synchrony of activated axons. These multi-parametric measurements provided a detailed and objective evaluation of peripheral nerve function.

### Joint range of motion measurement

Active range of motion (AROM) and passive range of motion (PROM) were measured in degrees (°) for all three groups before and after treatment using a standard goniometer.

### Pain assessment

Pain intensity was assessed for all three groups before and after treatment using the Global Pain Scale (GPS). This scale evaluates pain across four dimensions: clinical manifestations, emotional feelings, daily activities, and pain perception. It comprises 20 items, each rated on a scale from 0 (indicating minimal symptom severity) to 10 (indicating maximal symptom severity), with a maximum possible score of 50 points per dimension.

### Inflammatory cytokine levels

Fasting venous blood samples (4 mL) were collected from all participants between 7:00 and 9:00 a.m. after an overnight fast (≥ 8 h), both before treatment initiation and at the 60-day follow-up assessment. Samples were allowed to clot at room temperature for 30 min and then centrifuged at 3,000 rpm for 15 min at 4°C. The separated serum was aliquoted into sterile cryovials and immediately stored at –80°C until batch analysis to minimize freeze-thaw cycles. Serum concentrations of nuclear factor kappa B (NF-κB), tumor necrosis factor-alpha (TNF-α), and interleukin-6 (IL-6) were quantified using commercially available enzyme-linked immunosorbent assay (ELISA) kits (Shanghai Jianglai Biotechnology Co., Ltd., China), strictly according to the manufacturer’s instructions. All assays were performed in duplicate by laboratory technicians who were blinded to the clinical data and group allocation of the samples. The intra- and inter-assay coefficients of variation for all analytes were maintained below 10%.

### Statistical analysis

Statistical analyses were performed using SPSS software (version 22.0; IBM Corp., Armonk, NY, United States). Data were checked for normality using the Shapiro-Wilk test. All continuous data were normally distributed and are presented as mean ± standard deviation (x̄ ± s). Categorical data are expressed as frequencies and percentages (n,%). Comparisons of categorical variables among the three groups were performed using the chi-square (χ^2^) test. For ordinal efficacy data, the Kruskal-Wallis H test was used for overall comparison, followed by the Mann-Whitney U test for *post-hoc* pairwise comparisons between the combination therapy group and each monotherapy group. For continuous outcomes, *t*-tests were used to assess within-group changes from before to after treatment and to compare post-treatment outcomes between the combination therapy group and each monotherapy group. A two-tailed *P*-value < 0.05 was considered statistically significant for all tests. All analyses were conducted based on the intention-to-treat principle, and investigators remained blinded to group allocation throughout the statistical process to ensure objectivity.

## Results

### Comparison of therapeutic efficacy among groups

The baseline characteristics of the subjects in this study are presented in Supplementary Table 1. No significant differences were observed in the etiology of PNI, baseline severity, duration (acute/chronic), or potentially affected nerves. The combined treatment group (neurolysis + methylcobalamin) demonstrated a significantly higher total effective rate (86.67%) compared to the neurolysis-alone group (50.00%) and the methylcobalamin-alone group (53.33%) (Table 1).

**TABLE 1:** Comparison of efficacy among three groups [*n* (%)].

Group	Cured	Markedly	Effective	Ineffective	Total effective
Neurolysis	4 (13.33)	6 (20.00)	5 (16.67)	15 (50.00)	15 (50.00)
Mecobalamin	5 (16.67)	4 (13.33)	7 (23.33)	14 (46.67)	16 (53.33)
Combined	10 (33.33)	9 (30.00)	7 (23.33)	4 (13.33)	26 (86.67)
Z^A^					2.902
p^A^					0.004
Z^B^					2.794
p^B^					0.005

Z^A^, p^A^: Z and *P*-values compared between the neurolysis group and the combination group; Z^B^, p^B^: Z and *P*-values compared between the methylcobalamin group and the combination group. *P* < 0.05 indicates a statistically significant difference.

### Comparison of MCV, SCV, AMP, and LAT changes among treatment groups

Baseline MCV (Table 2), SCV (Table 3), AMP (Table 4), and LAT (Table 5) measurements showed no statistically significant differences among the neurolysis, methylcobalamin, and combination therapy groups (*P* > 0.05). Post-treatment evaluation revealed significant MCV, SCV, AMP, and LAT improvement in all three intervention groups compared to their respective pretreatment values (*P* < 0.05). Moreover, the combination therapy group exhibited significantly greater improvements than either monotherapy group in all four electrophysiological parameters, with post-treatment values showing statistically significant differences (*P* < 0.05; Tables 2–5).

**TABLE 2:** Comparison of MCV among three groups [m/s, (x ± s)].

Group	MCV		*t*	*p*
	Before treatment	After treatment		
Neurolysis	36.44 ± 3.90	42.60 ± 3.80	6.196	< 0.001
Mecobalamin	37.32 ± 4.19	43.66 ± 4.17	5.821	< 0.001
Combined	38.16 ± 3.38	49.46 ± 5.16	9.977	< 0.001
t^A^	1.814	5.863		
p^A^	0.075	< 0.001		
t^B^	0.841	4.788		
p^B^	0.404	< 0.001		

t^A^, p^A^: *t*-values and *P*-values compared between the neurolysis group and the combination group; t^B^, p^B^: *t*-values and *P*-values compared between the methylcobalamin group and the combination group. *P* < 0.05 indicates a statistically significant difference.

**TABLE 3:** Comparison of SCV among three groups [m/s, (x ± s)].

Group	SCV		*t*	*p*
	Before treatment	After treatment		
Neurolysis	33.60 ± 3.07	42.71 ± 3.66	10.433	< 0.001
Mecobalamin	34.67 ± 2.92	42.34 ± 3.48	9.180	< 0.001
Combined	34.04 ± 3.16	50.58 ± 5.00	15.232	< 0.001
t^A^	0.540	6.950		
p^A^	0.592	< 0.001		
t^B^	–0.789	7.406		
p^B^	0.433	< 0.001		

t^A^, p^A^: *t*-values and *P*-values compared between the neurolysis group and the combination group; t^B^, p^B^: *t*-values and *P*-values compared between the methylcobalamin group and the combination group. *P* < 0.05 indicates a statistically significant difference.

**TABLE 4:** Comparison of AMP among three groups [mV, (x ± s)].

Group	AMP		*t*	*p*
	Before treatment	After treatment		
Neurolysis	2.20 ± 0.34	4.01 ± 0.33	20.871	< 0.001
Mecobalamin	2.09 ± 0.31	4.72 ± 0.48	25.108	< 0.001
Combined	2.08 ± 0.36	5.06 ± 0.48	27.215	< 0.001
t^A^	1.366	9.873		
p^A^	0.177	< 0.001		
t^B^	0.224	2.745		
p^B^	0.823	0.008		

t^A^, p^A^: *t*-values and *P*-values compared between the neurolysis group and the combination group; t^B^, p^B^: *t*-values and *P*-values compared between the methylcobalamin group and the combination group. *P* < 0.05 indicates a statistically significant difference.

**TABLE 5:** Comparison of LAT among three groups [ms, (x ± s)].

Group	LAT		*t*	*p*
	Before treatment	After treatment		
Neurolysis	6.16 ± 0.92	5.23 ± 0.46	4.970	< 0.001
Mecobalamin	6.10 ± 0.94	4.42 ± 0.62	8.108	< 0.001
Combined	6.25 ± 0.89	4.06 ± 0.52	11.485	< 0.001
t^A^	0.370	9.167		
p^A^	0.713	< 0.001		
t^B^	0.590	2.430		
p^B^	0.557	0.018		

t^A^, p^A^: *t*-values and *P*-values compared between the neurolysis group and the combination group; t^B^, p^B^: *t*-values and *P*-values compared between the methylcobalamin group and the combination group. *P* < 0.05 indicates a statistically significant difference.

### Comparative analysis of AROM among treatment groups

As summarized in Table 6, the three treatment groups exhibited comparable baseline AROM values without statistically significant differences (*P* > 0.05). Post-intervention analysis revealed significant improvements in AROM across all groups compared to their pretreatment levels (*P* < 0.05), with the combination therapy group demonstrating the most pronounced enhancement. The mean AROM after treatment in the combination group was significantly higher than that in the neurolysis-alone group (*P* < 0.001) and the methylcobalamin-alone group (*P* < 0.001).

**TABLE 6:** Comparison of AROM among three groups [°, (x ± s)].

Group	AROM		*t*	*p*
	Before treatment	After treatment		
Neurolysis	10.68 ± 2.16	21.10 ± 3.65	13.462	< 0.001
Mecobalamin	10.02 ± 2.14	21.18 ± 3.24	15.631	< 0.001
Combined	10.54 ± 2.13	42.17 ± 5.17	30.94	< 0.001
t^A^	–0.244	18.249		
p^A^	0.808	< 0.001		
t^B^	0.926	18.852		
p^B^	0.358	< 0.001		

t^A^, p^A^: *t*-values and *P*-values compared between the neurolysis group and the combination group; t^B^, p^B^: *t*-values and *P*-values compared between the methylcobalamin group and the combination group. *P* < 0.05 indicates a statistically significant difference.

### Comparative analysis of PROM among treatment groups

Initial assessment revealed no significant between-group differences in PROM measurements (*P* > 0.05). Following therapeutic intervention, all three treatment groups (neurolysis, methylcobalamin, and combined therapy) demonstrated statistically significant improvements in PROM values compared to baseline (*P* < 0.05), as detailed in Table 7. Between-group comparisons showed that the combination therapy yielded significantly greater PROM gains than neurolysis alone (*P* < 0.001) and methylcobalamin alone (*P* < 0.001).

**TABLE 7:** Comparison of PROM among three groups [°, (x ± s)].

Group	PROM		*t*	*p*
	Before treatment	After treatment		
Neurolysis	24.86 ± 2.47	42.01 ± 3.38	22.468	< 0.001
Mecobalamin	24.57 ± 2.42	41.73 ± 4.01	19.948	< 0.001
Combined	24.20 ± 2.62	62.58 ± 5.33	35.257	< 0.001
t^A^	–0.989	17.850		
p^A^	0.327	< 0.001		
t^B^	–0.550	17.113		
p^B^	0.584	< 0.001		

t^A^, p^A^: *t*-values and *P*-values compared between the neurolysis group and the combination group; t^B^, p^B^: *t*-values and *P*-values compared between the methylcobalamin group and the combination group. *P* < 0.05 indicates a statistically significant difference.

### Comparative analysis of pain assessment scores across treatment groups

Pre-treatment evaluation showed no statistically significant differences among the three groups (neurolysis, methylcobalamin, and combination therapy) across four assessment domains: clinical manifestations, emotional status, daily activities, and pain intensity (*P* > 0.05). Post-treatment analysis revealed significant reductions in all measured domains compared to baseline values (*P* < 0.05). Notably, the combination therapy group achieved superior pain relief outcomes, demonstrating significantly lower scores than either single-treatment group (*P* < 0.05, Table 8).

**TABLE 8:** Comparison of pain scores among three groups [points, (x ± s)].

Group	Time	Clinical symptoms	Emotional feelings	Daily behavior	Pain
Neurolysis	Before treatment	35.39 ± 5.06	30.39 ± 5.45	26.74 ± 4.14	29.18 ± 5.33
	After treatment	22.70 ± 4.15*	17.68 ± 2.57*	17.58 ± 2.83*	18.49 ± 2.35*
Mecobalamin	Before treatment	33.29 ± 5.40	28.56 ± 4.90	27.29 ± 3.07	26.49 ± 4.47
	After treatment	20.63 ± 4.96*	18.35 ± 3.02*	18.75 ± 1.20*	19.03 ± 1.15*
Combined	Before treatment	33.78 ± 5.01	31.07 ± 5.22	26.91 ± 3.70	30.54 ± 4.46
	After treatment	13.74 ± 2.04*^#^	16.13 ± 2.46*^#^	13.27 ± 3.35*^#^	12.92 ± 1.85*^#^

Compared with before treatment in the same group,

*P < 0.05; Compared with Neurolysis or Mecobalamin after treatment,

#*P* < 0.05.

### Comparative effects on inflammatory cytokine levels among treatment groups

Pre-treatment serum concentrations of NF-κB, TNF-α, and IL-6 showed no significant intergroup differences (*P* > 0.05). Following treatment, all three groups exhibited significant reductions in these inflammatory markers compared to baseline levels (*P* < 0.05). The combination therapy group demonstrated significantly greater suppression of inflammatory cytokines than either neurolysis or methylcobalamin monotherapy (*P* < 0.05, Table 9). Additionally, after treatment, the incidence rates of limb numbness, joint stiffness, chronic pain, and limb atrophy in the combination group were significantly lower than those in either the neurolysis group or the methylcobalamin monotherapy group (*P* < 0.05), as shown in Table 10.

**TABLE 9:** Comparison of inflammatory cytokine levels among three groups (x ± s).

Group	Time	NF-κ B (μ g/L)	TNF-a (μ g/L)	IL-6 (pg/mL)
Neurolysis	Before treatment	28.25 ± 4.95	34.07 ± 5.04	16.32 ± 6.29
	After treatment	20.56 ± 3.90*	23.11 ± 3.77*	11.17 ± 3.20*
Mecobalamin	Before treatment	27.44 ± 5.12	34.14 ± 5.74	19.79 ± 5.73
	After treatment	18.40 ± 4.08*	18.25 ± 3.36*	10.74 ± 2.17*
Combined	Before treatment	27.87 ± 5.08	32.72 ± 5.09	18.67 ± 6.33
	After treatment	13.76 ± 2.10*^#^	16.45 ± 2.94*^#^	6.37 ± 1.56*^#^

Compared with before treatment in the same group,

*P < 0.05; Compared with Neurolysis or Mecobalamin after treatment,

^#^*P* < 0.05.

**TABLE 10:** Comparison of incidence of complications among three groups [n (%)].

Group	Limb numbness	Joint stiffness	Chronic pain	Limb atrophy
Neurolysis	16 (53.33)	17 (56.67)	22 (73.33)	18 (60.00)
Mecobalamin	12 (40.00)	16 (53.33)	19 (63.33)	15 (50.00)
Combined	3 (10.00)*^#^	2 (6.67)*^#^	3 (10.00)*^#^	4 (13.33)*^#^

Compared with Neurolysis,

*P < 0.05; Compared with Mecobalamin,

^#^*P* < 0.05.

## Discussion

PNI presents a complex clinical challenge, requiring interventions that address both mechanical compression and the underlying inflammatory pathophysiology (Sarhane et al., 2023). This study systematically compared the therapeutic efficacy of neurolysis alone, methylcobalamin alone, and their combination across multiple functional, electrophysiological, and biochemical parameters. The results demonstrate that the combined therapy not only significantly outperforms either monotherapy but also exhibits synergistic effects, providing comprehensive benefits for PNI patients.

Neurolysis is employed not only for managing both cancerous and non-cancerous joint pain (Matsumoto et al., 2023; Tay et al., 2025) but also for treating PNI (Wu et al., 2022). This is achieved by releasing nerve compression or removing scar tissue restricting nerve mobility. Methylcobalamin is commonly used to treat conditions arising from vitamin B12 deficiency, including peripheral nerve damage (Suzuki et al., 2017). However, the efficacy of combination therapy using neurolysis and methylcobalamin for PNI remains underexplored. The primary finding of this study is the superior total therapeutic efficacy of the combination group (86.67%) compared to neurolysis alone (50.00%) and methylcobalamin alone (53.33%). This striking difference underscores the complementary mechanisms of the two treatments. Neurolysis directly alleviates mechanical compression by releasing constrictive tissues, thereby restoring nerve mobility and blood supply (Bohyn et al., 2022). However, as observed in the neurolysis-alone group, surgical intervention alone may not fully address the intrinsic biochemical derangements that impede nerve regeneration. Conversely, methylcobalamin, through its neurotrophic properties, promotes axonal regeneration and myelin synthesis (Nishimoto et al., 2015) but may lack the mechanical decompression necessary for severe PNI cases. The combination of these therapies bridges this gap, addressing both extrinsic and intrinsic barriers to recovery. For example, a patient with severe carpal tunnel syndrome might experience immediate relief from neurolysis, but the addition of methylcobalamin could further accelerate nerve repair and reduce inflammation, leading to more sustained functional improvement.

In particular, the superior total efficacy observed in the combination group can be partly attributed to the synergistic interplay between enhanced nerve conduction and reduced inflammatory burden. Improved MCV and SCV reflect restored axonal integrity and myelination, which are essential for translating neural repair into functional gains (Tang et al., 2021). Simultaneously, the attenuation of pro-inflammatory cytokines such as TNF-α and IL-6 may have contributed to a more favorable microenvironment for axonal regeneration, thereby amplifying the functional benefits of neurolysis and methylcobalamin co-administration. In our study, all groups demonstrated significant improvements in both motor (MCV) and sensory (SCV) nerve conduction velocities, with the combination therapy group showing the most pronounced enhancement. Consistent with this, the combination group also exhibited the most favorable changes in complementary electrophysiological parameters: the greatest reduction in electromyography latency (LAT) and the greatest increase in amplitude (AMP). Shortened LAT indicates improved efficiency of signal transmission, while enhanced AMP reflects increased recruitment of functional nerve fibers or improved myelination. This suggests that while both neurolysis and methylcobalamin independently contribute to nerve function recovery (Morani and Bodhankar, 2010), their combined use yields additive or even synergistic effects across multiple electrophysiological domains. These improvements likely underpin the enhanced AROM and PROM observed in the combination group, as more efficient neural conduction facilitates better motor control and joint mobility. Moreover, the reduction in inflammatory markers such as NF-κB and IL-6 may have contributed to the preservation and regeneration of myelinated fibers, further supporting the observed electrophysiological gains. Thus, the synergistic effects of combined therapy extend across molecular, electrophysiological, and functional domains, reinforcing the interdependence of these outcomes.

AROM and PROM improvements further underscored the superiority of combination therapy (Gary et al., 2023). The combination group achieved the largest increases in both AROM and PROM. These results highlight the importance of restoring nerve gliding and reducing adhesions (via neurolysis) alongside promoting neural plasticity (via methylcobalamin). This functional improvement is not merely a consequence of mechanical release but also reflects the neuroregenerative and anti-inflammatory effects of methylcobalamin, which together enhance neural plasticity and motor re-education. The integration of these mechanisms underscores how combined therapy addresses both structural and biological barriers to recovery.

Pain management is a critical component of PNI treatment, as chronic neuropathic pain significantly impairs quality of life (Du et al., 2023). The combination group demonstrated the most substantial reductions in Global Pain Scale (GPS) scores (Garg et al., 2020) across all domains—clinical manifestations, emotional status, daily activities, and pain perception. This comprehensive pain reduction likely stems from the dual action of neurolysis, which removes the source of mechanical pain, and methylcobalamin, which modulates inflammatory mediators known to perpetuate neuropathic pain. The anti-inflammatory effects of the treatments were quantified through serum levels of NF-κB, TNF-α, and IL-6. While all groups showed reductions in these cytokines post-treatment, the combination therapy group achieved the most significant suppression. These findings align with the known anti-inflammatory properties of methylcobalamin, which inhibits NF-κB activation and downstream proinflammatory cytokines (Song et al., 2025; Xu et al., 2016). Simultaneously, neurolysis reduces mechanical stress on nerves, preventing the release of damage-associated molecular patterns (DAMPs) that trigger inflammation. Notably, the reduction in pain scores correlated with declines in serum TNF-α and IL-6 levels, suggesting that inflammation may be a key driver of neuropathic pain in PNI. By dampening this inflammatory response, methylcobalamin likely potentiated the analgesic effect of neurolysis, which physically removes nociceptive stimuli. This dual mechanism highlights the interconnected roles of biochemical and structural factors in pain modulation.

This study has several limitations. First, while efforts were made to standardize the surgical and rehabilitation protocols, subtle individual variations in surgical execution and patient adherence to rehabilitation are inherent to clinical practice. Second, the study encompassed a spectrum of PNI etiologies; although their distribution was balanced across groups, the relative efficacy of the interventions might vary for specific subtypes of nerve injury. Third, as a single-center trial, the generalizability of our findings may be limited by the specific patient demographics, surgical expertise, and institutional protocols of our center. Although this design ensured consistency in treatment delivery and assessment, it also means that the results may not be directly applicable to other settings with different patient populations or practice patterns. Fourth, the follow-up period was limited to 60 days. While this duration is adequate to capture significant early functional and electrophysiological recovery, it does not permit assessment of the long-term durability of treatment effects, the stability of regained function, or potential late complications (e.g., recurrence of symptoms, late-onset pain, or delayed nerve deterioration). Therefore, we acknowledge that our conclusions regarding the superiority of combination therapy are currently confined to short-term outcomes. To address these limitations, future multicenter studies with extended follow-up (e.g., 12 months or longer) are essential to validate our findings across diverse populations and injury subtypes, and to establish the long-term efficacy and safety of the combined neurolysis and methylcobalamin approach. In addition, research focusing on homogeneous injury groups and optimizing the dose and duration of methylcobalamin therapy would further refine clinical recommendations.

This study demonstrates that the combination of neurolysis and methylcobalamin offers a synergistic approach to PNI management, surpassing monotherapies in functional, electrophysiological, and inflammatory outcomes. Neurolysis creates a conducive environment for nerve recovery by eliminating physical barriers, while methylcobalamin enhances intrinsic repair mechanisms and dampens inflammation. The results support the integration of mechanical decompression and pharmacological neuroprotection in clinical practice.

## Data Availability

The raw data supporting the conclusions of this article will be made available by the authors, without undue reservation.
