# Supplementary material for: Synergistic efficacy of combined neurolysis and methylcobalamin in peripheral nerve injury: a randomized clinical trial
Source: Front Integr Neurosci. 2026 Apr 8;20:1747898. doi: 10.3389/fnint.2026.1747898 (PMC13099820; doi:10.3389/fnint.2026.1747898)
Supplement: Supplementary file 1 [file Data_Sheet_1.docx]

Supplementary table Baseline Characteristics of Subjects [n (%)]

| factor | Neurolysis | Mecobalamin | Combined |
| --- | --- | --- | --- |
| Etiology |  |  |  |
| Trauma | 8（26.67） | 12（40.00） | 7（23.33） |
| Oppression | 12（40.00） | 9（30.00） | 8（26.67） |
| Iatrogenic | 10（33.33） | 9（30.00） | 15（50.00） |
| Severity |  |  |  |
| Mild | 16（53.33） | 15（50.00） | 12（40.00） |
| Severe | 14（46.67） | 15（50.00） | 18（60.00） |
| Duration |  |  |  |
| Chronic | 12（40.00） | 17（56.67） | 14（46.67） |
| Acute | 18（60.00） | 13（43.33） | 16（53.33） |
| Affected nerves |  |  |  |
| Median nerve | 9（30.00） | 6（20.00） | 11（36.67） |
| Ulnar nerve | 12（40.00） | 13（43.33） | 9（30.00） |
| Radial nerve | 9（30.00） | 11（36.67） | 10（30.33） |
