# Supplementary figures and images for: Synergistic efficacy of combined neurolysis and methylcobalamin in peripheral nerve injury: a randomized clinical trial
Source: Front Integr Neurosci. 2026 Apr 8;20:1747898. doi: 10.3389/fnint.2026.1747898 (PMC13099820; doi:10.3389/fnint.2026.1747898)

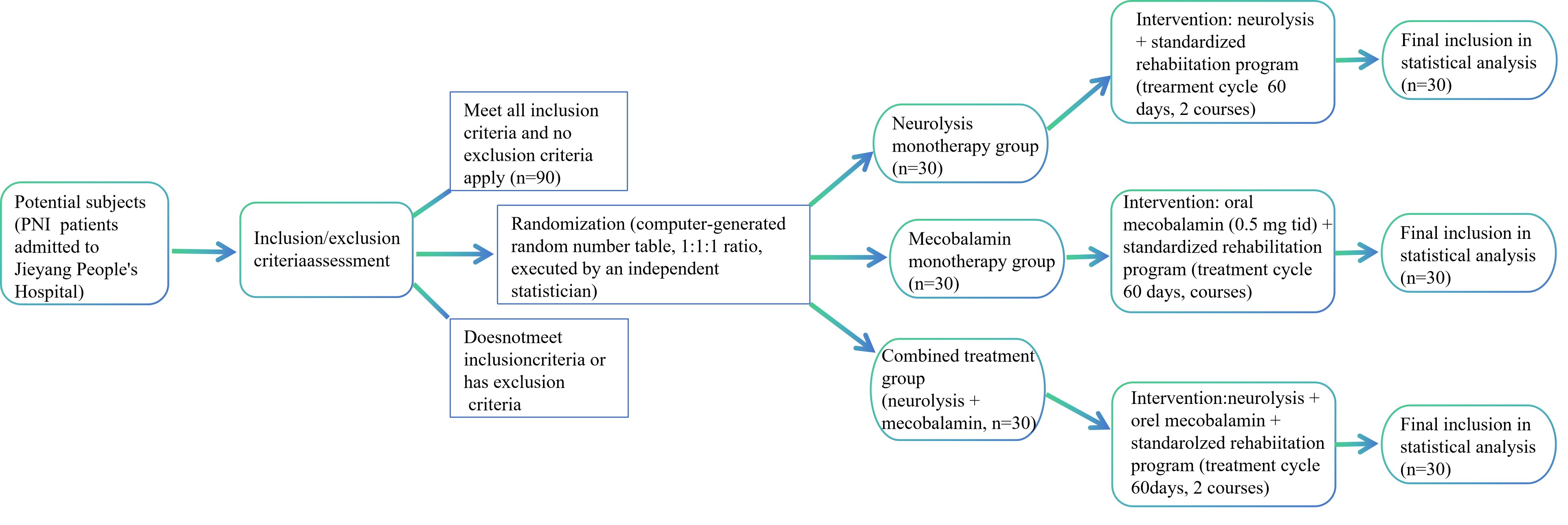

Supplement: Supplementary file 2 [file Image_1.jpeg]
